# The Anxious Mind: Examining the Conflicting Relationships Between Distinct Aspects of Social Anxiety and Social Cognition

**DOI:** 10.3390/bs16071228

**Published:** 2026-07-19

**Authors:** Shannon M. May Craig, J. Kiley Hamlin, Susan A. J. Birch

**Affiliations:** Department of Psychology, University of British Columbia, Vancouver, BC V6T 1Z4, Canada; kiley.hamlin@psych.ubc.ca (J.K.H.); sbirch@psych.ubc.ca (S.A.J.B.)

**Keywords:** social anxiety, social cognition, perspective-taking, Theory of Mind, emotion recognition

## Abstract

Social anxiety (SA) negatively impacts myriad aspects of an individual’s life. Although research with adults and children highlights an important link between SA and social cognitive abilities (e.g., reasoning about others’ thoughts and emotions), findings are mixed. We hypothesized that these mixed findings stem from the various combinations of social cognitive components of SA under investigation and the different types of measures used. Understanding these relationships in middle-to-late childhood is especially important, given that it is a period of substantial social cognitive development and a common onset age for SA. Seventy-eight children (*M*age = 8.15 years, *SD* = 1.61) and their parents completed measures capturing different components of anxiety (i.e., social worry, fear of negative evaluation, and social avoidance) and social cognition (i.e., emotion recognition, mental state understanding, and social perspective-taking). Measures of social cognition were not significantly correlated. Consistent with our expectations, associations between social cognition and social anxiety were measure-dependent. Self-reported fear of negative evaluation emerged as a positive predictor of accuracy in a behavioral measure of mental state understanding but a negative predictor of parent-reported mental state understanding. In addition, social avoidance accounted for additional variance only when predicting lower self-reported perspective-taking. Together, our findings highlight the multifaceted nature of social cognition and SA and the need for distinguishing these facets in future work. These findings are discussed in the context of measurement limitations in the current study and the related measurement challenges faced in this field.

## 1. Introduction

Social anxiety is characterized by a persistent and intense fear of social settings (Beck et al., 2005; Jefferson, 2001; Schneier & Goldmark, 2015; M. B. Stein & Stein, 2008). Critically, this fear is disproportionate to the situation and often detrimental to daily functioning, leading to extreme shyness, social withdrawal, and even panic attacks (Alomari et al., 2022). A clinical diagnosis of Social Anxiety Disorder (SAD) is met if symptoms persist for at least 6 months and cannot be explained by another disorder or medical condition (American Psychiatric Association, 2022).

Unfortunately, SAD and its symptoms are becoming increasingly common. In Canada and the US, lifetime prevalence is estimated to be 13% or higher among those aged 15 and older (Kessler et al., 2012; Stephenson, 2023). As an example, the number of Canadians diagnosed with anxiety and depression has more than doubled in the last 2 decades, and it was reported that the prevalence rates of SAD in adult Canadians increased 72% between 2002 and 2022 (Chau et al., 2026; Moroz et al., 2020). Childhood prevalence rates are notably lower, yet SAD is one of the most prevalent mental or emotional disorders in youth, with a global presence of up to 4.7% and 8.3% in children and adolescents, respectively (Salari et al., 2024). For comparison, the global prevalence of major depressive disorder has been estimated at 1.6% in children and 5.5% in adolescents (Lu et al., 2024). Age of onset occurs as early as preschool, though it more commonly emerges in middle childhood and adolescence (Baartmans et al., 2020; Halldorsson & Creswell, 2017; D. J. Stein et al., 2017; Stuijfzand & Dodd, 2017; Rapee et al., 2019). SAD is generally considered a form of internalizing psychopathology including symptoms of excessive fear, worry, and self-consciousness in social situations (Barajas-Gonzalez et al., 2020). SAD typically presents as behavioral inhibition in early childhood, often leading to more frequent internalizing symptoms (e.g., viewing themselves as unlikeable), heightening their fear of rejection, failure, and social evaluation (Ollendick & Hirshfeld-Becker, 2002). Unfortunately, SAD often develops into a chronic condition with severe and long-term consequences, such as poor mental health as well as dissatisfaction with and/or lower achievement in work, education, and relationships (Baartmans et al., 2020; Beidel & Alfano, 2011; Russell & Topham, 2012; Vilaplana-Pérez et al., 2021).

Symptoms of SAD vary widely among individuals and exist on a continuum ranging from severe—including incapacitating dread, panic attacks, and social withdrawal—to more moderate—including nervousness, worry, and gaze avoidance (Crişan et al., 2016; Dell’Osso et al., 2023; M. B. Stein et al., 2000; D. J. Stein et al., 2004). Importantly, social difficulties associated with social anxiety are not restricted to populations that meet the clinical threshold for a SAD diagnosis; research suggests that even subclinical symptoms, such as social discomfort or heightened emotional arousal, significantly impact quality of life (Davidson et al., 1994; Fehm et al., 2008; Filho et al., 2010; Kashdan & Roberts, 2004; Merikangas et al., 2002; M. B. Stein et al., 1994; Turner et al., 1986). For instance, both adults and children who exhibit social anxiety tend to be rated by their peers as less socially competent (Alden & Taylor, 2004; Meleshko & Alden, 1993), less likable, and less warm (Creed & Funder, 1998; Papsdorf & Alden, 1998). Perhaps unsurprisingly, then, socially anxious individuals are reported as having fewer close friendships and romantic relationships compared to the general population as well as those with other anxiety disorders (e.g., Generalized Anxiety Disorder, Alden & Taylor, 2004).

Subclinical symptoms of social anxiety at any point put individuals at higher risk of reaching clinical levels of anxiety and secondary depression in the future (Beesdo et al., 2007; Bosman et al., 2019; Zhong et al., 2024); thus, understanding the emergence of social anxiety in childhood is of particular importance. Indeed, positive and stable social relationships are essential for good health outcomes in children (Birrell et al., 2025; Larkins, 2014; Peters et al., 2011), whereas a lack of social connection is associated with negative mental and physical health problems as well as poorer cognitive development; that said, individual outcomes may show high variability (Almeida et al., 2021; Batty et al., 2021; Holt-Lunstad et al., 2010; Pearce et al., 2023). Failure to build stable social connections in childhood may lead to further social withdrawal, feelings of loneliness, and fewer opportunities to develop interpersonal skills, thereby exacerbating social anxiety symptoms (Ginsburg et al., 1998; Jefferson, 2001; Lacombe et al., 2024; Rapee et al., 2023; Reznick, 1989; Rubin & Mills, 1988). Although socially anxious individuals can experience peer difficulties at any age, they are particularly concerning in late childhood and adolescence, when peer acceptance and the presence of a supportive social network become increasingly crucial (Crone & Dahl, 2012; Rudolph, 2021).

Children’s social skills (or difficulties) are intimately tied to their social cognitive development (i.e., the range of cognitive processes which enable people to function within a social group) (Frith, 2008). In the current manuscript, we are specifically interested in the varied social cognitive processes involved in identifying and reasoning about the mental and emotional states of others, sometimes called ‘social perspective-taking’ or ‘mental state understanding’. Importantly, certain aspects of these social cognitive processes have been linked to clinical and subclinical levels of social anxiety in both adults and children (Alvi et al., 2022; Briscoe et al., 2024; Eikelboom et al., 2025; Huang et al., 2026; Karaca Cengiz et al., 2025; McClure & Nowicki, 2001), but considerable debate remains over exactly which social cognitive processes are affected and how.

A large body of research in adults suggests that social anxiety is associated with poorer social cognition. For instance, studies show that socially anxious individuals often perceive ambiguous social cues as more negative or threatening, such as mislabeling neutral faces as angry (Alden et al., 2008; Beck et al., 2005; Gutiérrez-García & Calvo, 2017; Heuer et al., 2010; Maoz et al., 2016; Peschard & Philippot, 2017; Yoon et al., 2014, Yuan et al., 2025b). They may also have difficulty disengaging from negative social stimuli, like a look of disgust or a fearful tone of voice (Clark & Wells, 1995; Rapee & Heimberg, 1997). Other findings suggest that socially anxious individuals avoid engaging with negative social stimuli in the first place and instead show an inappropriate amount of attention to the self and their internal states (Adamis et al., 2025; Mansell et al., 2003; Mellings & Alden, 2000; Poole & Henderson, 2022; see also Crozier, 1979 and Melchior & Cheek, 1990) that can lead to slower reaction times and poorer accuracy in identifying social cues (Tseng et al., 2017). Altogether, these findings suggest that biases in social attention and interpretation are heightened in socially anxious individuals. This may lead them to misinterpret, discount, or exaggerate important social feedback, resulting in poorer social cognition and limited capacity to reason about the mental states of others (Amir et al., 2003; Buckner et al., 2010; Liang et al., 2017; Liu et al., 2024; Vytal et al., 2013).

In contrast to the work reviewed above, some research shows no significant differences between socially anxious adults and healthy controls in various social cognitive tasks, including accuracy-based (de Jong & Martens, 2007; Folz et al., 2023; Lenton-Brym et al., 2018; Reinholdt-Dunne et al., 2009; Torro-Alves et al., 2016), interview, and self-report measures (Ballespí et al., 2021; Nunes et al., 2018; for a meta-analysis see Pittelkow et al., 2021) of both emotion recognition and mental state reasoning. In contrast, other studies have demonstrated that socially anxious individuals are actually *better* in some social cognitive tasks, such as recognizing others’ emotional states or detecting changes in the facial expressions of others (Arrais et al., 2010; Joormann & Gotlib, 2006; Yuan et al., 2025a), perhaps because socially anxious individuals develop a heightened sensitivity to the thoughts and feelings of others (Tibi-Elhanany & Shamay-Tsoory, 2011).

How do we reconcile these conflicting results? Previous studies (i.e., those outlined above as well as several examples below) have used a wide range of study designs to investigate the relationship between social anxiety and social cognition. The seemingly mixed findings likely reflect heterogeneity in the aspects of social anxiety under investigation, in the components of social cognition under investigation, as well as differences in the types of measures employed to assess each construct. For example, levels of social anxiety are often evaluated based on ‘cognitive’ symptoms, such as feelings of social worry or nervousness about social settings and/or the fear of negative evaluation (Gao et al., 2025; Wong et al., 2016). On the other hand, social avoidance and social withdrawal are behavioral symptoms (Rodebaugh et al., 2025). Unfortunately, because previous research has not consistently differentiated between cognitive and behavioral components of social anxiety, it is difficult to understand their (potentially unique) relationships with social cognition.

Adding to the complexity, studies vary in which components of social cognition they measure and how they measured them. For instance, emotion recognition, argued to be a relatively basic component of social cognition (Malle, 2021), relies on perception and attention and involves recognizing observable facial and prosodic (i.e., vocal) cues (Brothers & Ring, 1992; Ochsner, 2008; Frith & Frith, 2006; Fitzpatrick et al., 2018; Singer, 2006). In comparison, social perspective-taking or mental state understanding requires making inferences and reasoning about broader, more complex, and largely unobservable mental states, including people’s beliefs, desires, knowledge, intentions, and hidden or ‘masked’ emotions (Corrigan, 1997; Malle, 2021).

A third complication involves the considerable variability in the types of tasks used to assess different components of social cognition. This variation may account for, or at least contribute to, the mixed results regarding their association with social anxiety. For example, some behavioral tasks measure *accuracy* in mental state reasoning or emotion recognition (e.g., False Belief tasks or the Reading the Mind in the Eyes task; Baron-Cohen et al., 2016; Bauminger-Zviely, 2013), while others are self-report measures asking informants to *subjectively* reflect on their own (or someone else’s) social cognitive capacity (e.g., Interpersonal Reactivity Index or the Four Item Mentalizing Index; Davis, 1980; Clutterbuck et al., 2021). Scores from these different tasks may have distinct relationships with social anxiety. On the one hand, some research suggests that socially anxious individuals tend to ‘overmentalize’ in certain social situations, attributing more thoughts or intentions to another person than appropriate. For example, attributing a person’s silence to anger or quiet resentment rather than fatigue. Therefore, socially anxious individuals may report *thinking about* mental states more frequently (Öztürk et al., 2022; Pittelkow et al., 2021; Tibi-Elhanany & Shamay-Tsoory, 2011) but nevertheless be less *accurate* when doing so (Ballespí et al., 2019, 2021; see also Hunsche, 2025, for an interesting exception in individuals with autism spectrum disorder with comorbid social anxiety). On the other hand, individuals who ‘undermentalize’ (i.e., fail to notice or reason about mental states) in social situations, perhaps due to greater anxiety and avoidance (Chevalier et al., 2023), may rate themselves lower on self-report measures of mental state reasoning but perform accurately in certain lab-based tasks where the social stimuli are more restricted or controlled. Thus, self-report measures are especially prone to confounding an individual’s *accuracy* in mental state understanding and their *propensity*, or tendency, to notice and reason about mental states.

Importantly, research examining the relationship between social anxiety and social cognition in children, though less extensively studied, mirrors the mixed results observed with adults. Some studies indicate poorer performance in socially anxious children, such as mislabeling emotional cues (Battaglia et al., 2004; McClure & Nowicki, 2001); other studies show no difference (Mobach et al., 2022; Pequet & Warnell, 2021) or indicate greater motivation and precision in processing social information (Ale et al., 2010; Pequet & Warnell, 2021). For example, Ale and colleagues (2010) reported that children experiencing social anxiety are more vigilant in social settings and more accurate in labeling facial expressions. In contrast, in one of the few studies examining prosodic emotion recognition, McClure and Nowicki (2001) found that children aged 8 to 10 with higher levels of social anxiety and social avoidance were more likely to mislabel fearful and sad emotional cues. Notably, however, a *prosodic* emotion recognition task was used, requiring children to identify emotions using only vocal cues. Given the absence of facial expressions, this type of emotion recognition task was arguably harder and more likely to capture subtle difficulties in socially anxious and avoidant children. The idea that deficits in emotion recognition might be more pronounced when examining complex rather than basic emotions is consistent with previous studies examining children with ASD (Rodgers et al., 2021) as well as children with higher levels of social anxiety (O’Toole et al., 2013).

Mixed results as to the link between social anxiety and social perspective-taking arise not only from the *task* and its level of complexity, but also from the *target* whose perspective is being taken. For instance, work has shown that children with higher social anxiety are significantly more likely to use mental state terms when describing a close friend (e.g., talking about their friend’s beliefs and preferences rather than their actions or physical appearance) than low-anxious people, as compared to an acquaintance, which could indicate a link between social anxiety and a heightened focus on the thoughts and feelings of close others (Pequet & Warnell, 2021). However, when asked to identify the emotions or mental states of a hypothetical stranger (e.g., using standard tasks like Happe’s Strange Stories and the Reading the Mind in the Eyes task), social anxiety did not relate to children’s perspective-taking performance (Pequet & Warnell, 2021).

Altogether, the mixed results outlined above suggest that a complete understanding of how social cognitive processes are related to social anxiety will require a multifaceted and multi-measure approach to measuring both social cognition and social anxiety. We anticipate that the conflicting findings arise from the inconsistency in measures used, as well as genuine differences in the constructs being measured. The primary goal of the current study was to gain a clearer and more comprehensive understanding of the nature of the relationships between different components of social cognition and different aspects of social anxiety. The present study recruited children aged 6 to 12 for numerous reasons. First, middle-to-late childhood is a common onset age for social anxiety and a time period that coincides with entering school, when SA can particularly impact a child’s daily functioning (Halldorsson & Creswell, 2017; Halldorsson et al., 2023; Ollendick & Hirshfeld-Becker, 2002). Second, the elementary period is when children make considerable progress in mental state understanding alongside increasing consideration of others’ mental states (Fu et al., 2023). Thus, this window of intense social cognitive development, combined with the emergence of significant SA symptomatology, has the potential to offer unique insights into how aspects of social anxiety interact with various social cognitive processes. Finally, research suggests a slight advantage in social cognitive development in girls throughout childhood (Baron-Cohen, 2002), as well as higher rates of social anxiety in girls (Asher et al., 2017; Essau et al., 1999; Merikangas et al., 2010). Therefore, examining this period may provide insight into the role of gender in the relationships between social cognition and social anxiety.

The current study implemented a multi-measure cross-sectional design with 6–12-year-olds, as better elucidating the relationship between SA and social cognition within this developmental window requires examining whether and to what degree different aspects of social cognition and social anxiety are related in individual children. Although a cross-sectional approach does not allow us to pinpoint the causal nature of any observed relationships, it helps to pave the way for future work that does. Equally importantly, this work will shed critical light on the direction and magnitude of the distinct relationships between different components of social cognition and different aspects of social anxiety. Children’s social cognition was measured using two behavioral measures examining social cognitive *accuracy*: one focusing on mental state understanding and the other on emotion recognition. In addition, we utilized a self-report (subjective) measure of children’s *tendency* to engage in social perspective-taking. Finally, children’s parents completed a measure of their perception of their child’s mental state understanding and mentalizing tendencies, as revealed by their day-to-day behaviors.

Importantly, this design allowed us to assess whether and how different aspects of children’s social cognitive abilities relate to one another. We investigated different components of children’s social cognitive abilities using four different measures of social cognitive processes, focusing on those most commonly used in research comparing social anxiety and social cognition (i.e., emotion recognition and mental state understanding; see Baez et al., 2023). No previous research has used these specific social cognition measures together. In the absence of such data, we hypothesized that the four measures of social cognitive ability would be modestly correlated given the broad nature of social cognition and the distinct components and measurement types being investigated.

Children’s social anxiety was also measured in multiple ways. The current study used child- and parent-report questionnaires to measure children’s levels of general social worry, social avoidance, and fear of negative evaluation. Parent-reports were utilized so that we could examine the correlation between these and children’s self-reports; however, given that prior studies find that children over the age of five can reliably report on their emotional and social well-being (Caqueo-Urízar et al., 2022; Kiel et al., 2015; Varni et al., 2007), and that children are in a better position to report on their internal states than their parents, we preregistered that wherever our child- and parent-reports were redundant (i.e., social worry, social avoidance, and fear of negative evaluation), we would utilize child-reports in our analyses so long as they correlated moderately well with parent-reports.

Akin to our predictions regarding social cognition, we expected moderate correlations between different aspects of social anxiety. Social worry, fear of negative evaluation, and social avoidance are all considered key factors of social anxiety whose measures are often combined into one overall score of social anxiety (Fresco et al., 2001; Watson & Friend, 2012; Wong & Rapee, 2016). Nonetheless, we did not expect to find overly strong correlations, as they are distinct aspects of social anxiety and may vary from person to person (e.g., Gao et al., 2025; Rodebaugh et al., 2025; Wong et al., 2016).

Critically, in the present study, we hypothesized that the relationships between social cognition and social anxiety would be conceptually distinct and measure-dependent. Specifically, we expected that cognitive symptoms of social anxiety would predict *lower* scores on three specific components of social cognition, including emotion recognition as well as self- and parent-reported mental state understanding, but *higher* scores in self-reported social perspective-taking, reflecting a heightened sensitivity to others’ thoughts and feelings as observed in prior research (Öztürk et al., 2022; Tibi-Elhanany & Shamay-Tsoory, 2011).

We also predicted that the behavioral aspect of social avoidance would account for additional variability in each case, consistent with the theory that social avoidance plays an especially impactful role in the relationship between social anxiety and social cognition as well as the development of social competency (Ghandchi et al., 2023; McClure & Nowicki, 2001; Stevens et al., 2014). Interestingly, although social avoidance is less common than general social worry or fear of negative evaluation (Chen et al., 2020), it may also be particularly detrimental to social cognitive ability, in that avoiding social interactions limits opportunities to learn about others’ mental states. Indeed, research has identified social avoidance as a significant predictor of future social anxiety, and some researchers consider it a central feature in the maintenance of SAD as well as the most likely to affect the processing of social cues (Hodson et al., 2008; Leigh & Clark, 2018; McClure & Nowicki, 2001; Rodebaugh et al., 2025; Schreiber et al., 2012; Stevens et al., 2014).

Finally, we controlled for age and gender in our analyses. Given there is substantial social cognitive development (Fu et al., 2023), as well as greater social awareness and peer evaluation within this age range (Poole & Henderson, 2022), we predicted that age would positively correlate with children’s overall accuracy in social cognitive tasks and levels of social anxiety. In addition, we expected to find a small advantage for girls in children’s social cognitive performance as well as heightened social anxiety in girls, irrespective of age and consistent with the prior literature (Asher et al., 2017; Baron-Cohen, 2002; Essau et al., 1999; Merikangas et al., 2010).

## 2. Materials and Methods

### 2.1. Participants

A total of 78 children (*M* = 8.15 years, *SD* = 1.61 years; 64.1% female, 2.6% missing gender) and their parents participated in this study. Although 97 participants were recruited, 19 were excluded for failure to complete at least 80% of items on each measure. Inclusion criteria included being, or being the primary caregiver of, a child aged 6–12, as well as being sufficiently fluent in English (defined as English being the primary language used at least 50% of the time). Our sample would be classified as an ‘unselected’ sample, rather than clinical or non-clinical, as participants were neither selected nor disqualified based on their psychopathology or clinical diagnosis.

Parent participants were 80.8% female (5.1% opted not to respond), and the majority identified as East Asian (48.7%) or European (29.5%). The remaining participants reported being East Indian (5.1%), Middle Eastern (2.6%), Latin/Central/South American (1.3%), mixed (2.6%), or other (3.8%); 6.4% opted not to respond. The sample was generally highly educated (80.7% of parents completed a bachelor’s degree or higher), and of mid-to-high economic status (e.g., 52.6% reported a family income of 100,000 per year or greater).

### 2.2. Measures

#### 2.2.1. Social Cognition Measures

Prosodic Emotion Recognition: Children’s accuracy in prosodic emotion recognition (i.e., emotions conveyed through tone of voice) was measured using items from the Cambridge Mindreading Face–Voice Battery for Children (CAM-C; Golan et al., 2015). The CAM-C is a child-friendly adaptation of an adult battery designed to assess recognition of both simple and complex emotions in faces and voices. The child version uses static facial images (for ‘face trials’) and dynamic voice recordings (for ‘voice trials’) and has been validated for use with children aged 6–12, with a test–retest reliability for faces and voices ranging from 0.76 to 0.81 and an internal consistency of 0.74 for complex voices (Rodgers et al., 2021).

As mentioned above, previous studies have also shown that deficits in emotion recognition are more pronounced in studies using complex emotions (Rodgers et al., 2021) and more strongly related to social anxiety (O’Toole et al., 2013). Accordingly, the current study used a total of 10 items from the CAM-C that measured recognition of complex emotions conveyed solely through tone of voice, excluding items that were limited to basic emotions. We selected five complex vocal emotions that showed good reliability in previous studies in differentiating between typically developing children and those who struggle with social information processing, including *unfriendly*, *amused*, *nervous*, *bothered*, and *jealous* (Rodgers et al., 2021). Finally, for brevity, we selected two (of three) items for each complex emotion based on item-total correlations in past work (Rodgers et al., 2021). In the current sample using only these 10 items, Cronbach’s alpha was lower than previous work at 0.51.

The Theory of Mind scale (TOM scale): Children’s accuracy in understanding mental states was evaluated using 2 items from the 5-item version of the Theory of Mind scale (Wellman & Liu, 2004). In its original format, the TOM scale utilizes five tasks to evaluate a child’s understanding of others’ mental states, including desires, beliefs, knowledge, and emotions, particularly when these differ from the child’s own perspective or from objective reality. Wellman & Liu’s analyses revealed a clear developmental progression in children’s mental state understanding, such that items targeting simpler mental states showed ceiling effects past the age of six (see also Peterson et al., 2012). Therefore, the current study selected only the two most difficult tasks targeting complex mental states (i.e., Contents False Belief and Hidden Emotions). This measure was scored proportionally, meaning that participants’ scores reflected the proportion of Theory of Mind tasks they answered correctly (i.e., 0%, 50%, or 100%). Consistent with the scoring approach used by the authors in the original validation study, participants were required to pass a ‘memory’ check in the Contents False Belief task ensuring they paid attention and remembered the critical details of the story to have the trial included.

Children’s Social Understanding Scale (CSUS): The CSUS is a parent-report measure designed to evaluate a child’s understanding of others’ mental states, such as their beliefs, knowledge, desires, perceptions, and emotions by having parents report on a variety of behaviors they can observe in their child day-to-day. The current study used the short-form of the CSUS, which has been validated in children ages 2–7 and demonstrated high test–retest reliability (*r*(29) = 0.88) and internal consistency (α = 0.89), as well as positive correlations with two False Belief measures (including the Contents False Belief task used in the current study and one other) adapted from Wellman and Liu’s TOM scale (Tahiroglu et al., 2014). The CSUS-Short Form has been used with children up to age 12 and reliably predicts multiple facets of social-emotional health (Haddock & Birch, 2024). Cronbach’s alpha in our sample was high (α = 0.81).

Four Item Mentalizing Index (FIMI): The Four Item Mentalizing Index (FIMI) is a 4-item self-report measure of one’s tendency to engage in social perspective-taking (i.e., discerning the mental states of others within dynamic social contexts). It was originally validated in typically developing American adults (Clutterbuck et al., 2021) but has also shown cross-cultural reliability (Fekih-Romdhane et al., 2024). Previous analyses reported acceptable reliability for the FIMI, with a coefficient omega (ω) between 0.70 and 0.75 (Clutterbuck et al., 2021; Montgomery et al., 2024). As no validated self-reported measure of children’s perspective-taking exists in the literature for our target age range, we modified the adult self-report measure for use as both a parent- and child-report measure. For example, we created a child-report FIMI by substituting more child-friendly wording (e.g., *“I can usually understand another person’s viewpoint, even if it differs from my own”* was changed to *“I can understand what others think even if it’s not the same as what I think”*). In our sample, the coefficient omega for the child-report was moderately low at 0.53, indicating limited reliability, while the parent-report showed high reliability at 0.82.

#### 2.2.2. Social Anxiety Measures

Social Worry (SWAIY-modified): Social worry, a cognitive component of social anxiety, was measured using a modified version of the Social Worries Anxiety Index for Young Children (SWAIY), a 10-item parent-report measure modified from the Social Worries Questionnaire (SWQ; Spence, 1995) to assess anxiety in children aged 4–8 (Stuijfzand & Dodd, 2017). To ensure that our measure was appropriate for the full age range of our sample, we used all 10 items from the SWAIY, plus two additional items from the SWQ appropriate for older children (i.e., “*Gets worried about eating in public*” and “*Gets worried about using public toilets*”). Finally, the wording was changed to reflect a child-report rather than a parent-report. This left us with a final set of 12 items (SWAIY-modified; see Supplementary Materials—Section S1), with answers ranging from 0 (*Not at all like me*) to 2 (*A lot like me*). The N/A option from the original survey was removed to ensure the highest amount of usable data.

Social Avoidance (SWAIY-avoidance): Social avoidance scores, a behavior component of social anxiety, were measured by removing the word avoid in each item of the original SWAIY and adapting it into a new ‘yes’ or ‘no’ follow-up question. For example, the item *“Avoids or gets worried about going to parties or play dates”* became *“I get worried about going to parties or play dates”* to create a two-part question: the first assessing the child’s level of social worry about parties or playdates and the second asking a yes or no question about whether that social worry would lead them to avoid the situation altogether, (e.g., ‘avoid it all together?’, SWAIY-avoidance; see Supplementary Materials—Section S1). This allowed us to examine levels of social avoidance separately from social worry and to determine whether social avoidance impacts social cognitive processes independently from worry alone.

The 10-item SWAIY has shown high internal consistency (>0.80), test–retest reliability (*r* = 0.87), and convergent validity (*r* > 0.50) (Spence, 1995). In our sample, Cronbach’s alpha was 0.79 for the parent-report and 0.77 for the child-report for the 12 items measuring social worry. For the follow-up question on social avoidance (SWAIY-avoidance), Cronbach’s alpha was 0.78 for the parent-report and 0.47 for the child-report. To alleviate concerns regarding shared method variance and multicollinearity, we ensured the correlation between these two constructs did not exceed the recommended threshold of *r* = 0.80 (Mason & Perreault, 1991; de Winter, 2025). The correlation between these two measures was *r* = 0.24.

Fear of Negative Evaluation (FNE): Levels of social anxiety were also assessed through parental reports of children’s fear of being negatively evaluated in social settings, using the Brief Fear of Negative Evaluation scale (BFNE; Leary, 1983). A child-report version was created by simplifying the language to accommodate participants’ age and comprehension level (e.g., “*I am frequently afraid of other people noticing my shortcomings*” became “*I am afraid that other people will notice my mistakes*”).

In previous work, the BFNE scale’s test–retest reliability (ICC = 0.71) and Cronbach’s alpha (α = 0.82) for socially phobic students were both high (Tavoli et al., 2009). In our sample, Cronbach’s alpha was 0.93 for the parent report and 0.88 for the child report.

A summary of measures used in the current study, including scoring procedures and examples items, is presented in Table 1.

### 2.3. Procedure

Participants were recruited online through social media (Facebook and Instagram), posting the study’s promotional material, and a local database of interested families from the greater Vancouver area. All communication with parents before the study session was done via email. Once participation was confirmed and a child’s session was scheduled, parents received online consent forms for themselves and their child, as well as the parent-report survey to complete prior to their child’s participation. Child participation was done over Zoom (Zoom Communications Inc., San Jose, CA, USA) with a trained female researcher in a 30–45 min Zoom session. This study was conducted in the same session as a separate study (see OSF for separate study; 10.17605/OSF.IO/AMGX6); therefore, brevity in measures was an important consideration. Consent to participate and be recorded via Zoom was re-confirmed before the researcher began the study.

Researchers were provided with a script to maintain consistency between sessions. Children were asked to answer questions about different scenarios or indicate on a Likert-scale how much certain statements sounded like them (see Supplementary Materials—Section S1. Items and scenarios for each scale were read aloud. Participants were told they could stop their session at any time and skip any questions that they did not want to answer.

To minimize potential fatigue effects and maintain younger children’s attention and engagement throughout the study, we introduced intermittent “prizes” (e.g., coloring pages and activity worksheets that they selected to receive at the end of the study). We also told children they could stop at any time and approximately 75% of the way through the study, children were asked if they would like to stop or continue. All participants included in our study opted to continue; therefore, fatigue effects were likely minimal. At the end of the session, parents and their children were given the opportunity to provide feedback on the study and to choose between receiving a $10.00 Amazon gift card or entering a draw (1 in 25 chance) to win a $50.00 gift card as compensation.

### 2.4. Ethics Approval

This study was approved by the UBC Behavioral Research Ethics Board.

### 2.5. Statistics Used: Power Analysis, Correlations, and Regressions

We used correlations and hierarchical regressions to test our hypotheses. An a priori power analysis was conducted using GPower version 3.1.9.4 (Faul et al., 2009) to determine the required sample size for detecting a medium effect in a hierarchical regression with three predictors and two control variables (two-tailed, α = 0.05, power = 0.80). Our minimum target sample size was 77 participants to detect a medium effect.

### 2.6. Analytic Plan

We planned four regressions based on our a priori hypotheses, one for each component of social cognition. Since these models evaluated what we expected to be distinct components of social cognition, they were treated as separate inferences. We set our *p*-value for these confirmatory analyses at *p* = 0.05. Full details of our analytic plan were preregistered on the Open Science Foundation (see https://osf.io/muxvn/; accessed on 14 July 2025).

## 3. Results

### 3.1. Preliminary Analyses

Participant responses were summed for each of our measures, with higher scores indicating greater levels of social anxiety and better performance on social cognitive measures. One exception was the TOM scale, for which total scores were calculated using the proportion of tasks that participants passed both the attention check and the mental state understanding probe. We calculated means, standard deviations, and maximum scores for each variable (see Table 2).

#### 3.1.1. Age

We computed zero-order correlations between age and gender and our measures of social cognition and social anxiety to test the expected positive relationships with girls and older participants (see Table 3). As predicted, age was significantly correlated with higher accuracy on the two behavioral social cognition tasks (prosodic emotion recognition, *r*(76) = 0.38, *p* ≤ 0.001; the TOM scale, *r*(76) = 0.25, *p* = 0.026). However, against expectations, age did not correlate with either child- or parent-reported social cognitive ability (i.e., FIMI, CSUS).

Age was also positively correlated with most of our cognitive measures of social anxiety, though this correlation only reached significance in the child-report measure of the fear of negative evaluation, *r*(76) = 0.14, *p* = 0.032, and the parent-report measure of social worry, *r*(72) = 0.26, *p* = 0.022. No age differences emerged in the measure of social avoidance.

#### 3.1.2. Gender

Only one significant relationship emerged with gender: The parent-report measure of children’s tendency to engage in social perspective-taking (FIMI) was positively correlated with gender, indicating that parents reported their daughters were better able to take others’ perspectives than their sons, *r*(70) = 0.24, *p* = 0.044. Critically, however, children themselves did not show or report any gender differences in either mentalizing or social anxiety.

#### 3.1.3. Child- and Parent-Report Correlations

Next, we compared child- and parent-reports for our measures of social anxiety and found that all measures, including social worry, *r*(71) = 0.26, *p* = 0.027, social avoidance, *r*(53) = 0.30, *p* = 0.025, and the fear of negative evaluation, *r*(72) = 0.28, *p* = 0.015, were significantly and positively correlated. We used the child reports in our main analyses, as the correlations between child- and parent-reports either met or were very close to our predetermined threshold. These modest correlations between children- and parent- reports are consistent with the broader literature on children’s mental health (e.g., Caqueo-Urízar et al., 2022; De Los Reyes & Kazdin, 2005). For comprehensiveness and comparisons, we also conducted the main analyses using the parent-report data (see Supplementary Materials—Section S2). Results from these supplemental analyses generally followed the same pattern with exceptions noted within.

We also compared the child- and parent-report measures of social perspective-taking (FIMI) and found no relationship, *r*(70) = 0.03, *p* = 0.83. Nonetheless, we again chose to use the child-report in our main analyses, both to maintain consistency and because of the internalized and subjective nature of the constructs of interest.

#### 3.1.4. Assumption Checks

Measures were evaluated to ensure that they did not violate the assumptions of normality and homoscedasticity. A visual-based assessment of Q-Q plots suggested adequate normality; however, there was evidence of heteroscedasticity, specifically in the TOM scale and the CSUS parent-report. Thus, HC3 heteroscedasticity-consistent standard errors and 95% confidence intervals were computed using the “sandwich” package (Zeileis, 2004) in R version 4.6.1, following the HC3 approach described by Long and Ervin (2000). In addition, multicollinearity among predictors was evaluated in SPSS version 31 (IBM corp., Armonk, NY, USA) using the variance inflation factor (VIF) values. All VIF values were well below the conservative threshold of 5, indicating low risk of multicollinearity (see Supplementary Materials for VIF values—Section S4).

### 3.2. Main Analyses

#### 3.2.1. Correlations Between Social Cognitive Processes

We computed partial correlations between our four social cognitive variables to examine the degree of association between prosodic emotion recognition, mental state understanding, and self-reported social perspective-taking. We found little-to-no overlap between these measures, after controlling for age in months (see Table 4).

#### 3.2.2. Mental State Understanding (CSUS) Predicted by Social Anxiety

Hierarchical regression analyses predicting mental state understanding as assessed by the CSUS parent-report (Table 5) revealed that age and gender accounted for approximately 2.2% of its variability, *F*(2, 70) = 0.80, *p* = 0.45. Introducing levels of social anxiety—assessed through levels of social worry and fear of negative evaluation—accounted for an additional 6.7% of the variation in children’s mental state understanding (CSUS), and this change in *R*^2^ was not significant, *F*(1, 66) = 0.59, *p* = 0.446. Adding levels of social avoidance accounted for only an additional 0.8% of children’s mental state understanding (CSUS), and this change in *R*^2^ was not significant, *F*(1, 66) = 0.59, *p* = 0.446.

Furthermore, fear of negative evaluation (BFNE) emerged as a unique and significant predictor of mental state understanding, as assessed by the CSUS parent-report measure. A higher fear of negative evaluation was associated with lower ratings of parent-reported mental state understanding, *t*(68) = −2.05, *B* = −0.33, SEHC3 = 0.14, 95% CI [−0.61, −0.04], *p* = 0.044.

#### 3.2.3. Mental State Understanding (2-Item TOM Scale) Predicted by Social Anxiety

Hierarchical regression analyses predicting mental state understanding as assessed by the TOM scale (Table 6) revealed that age and gender accounted for approximately 9.7% of its variability, *F*(2, 72) = 3.85, *p* = 0.026. Introducing levels of social anxiety—assessed through levels of child-reported social worry and fear of negative evaluation—accounted for only an additional 5.0% of the variation in children’s mental state understanding (TOM scale), and this change in *R*^2^ was not significant, *F*(2, 70) = 2.07, *p* = 0.134. Adding levels of social avoidance accounted for zero additional variation in children’s mental state understanding (TOM scale).

Furthermore, in Model 3, child-reported fear of negative evaluation (BFNE) again emerged as a unique and significant predictor of mental state understanding as assessed by the TOM scale, with a higher fear of negative evaluation associated with greater accuracy in mental state understanding, *t*(68) = 2.17, *B* = 0.32, SEHC3 = 0.12, 95% CI [0.08, 0.57], *p* = 0.033. Notably, this relationship is in the opposite direction of that between the fear of negative evaluation and the CSUS as reported above.

#### 3.2.4. Social Perspective-Taking (FIMI) Predicted by Social Anxiety

Hierarchical regression analyses predicting child-reported social perspective-taking (Table 7) revealed that age and gender accounted for 1.4% of its variability, *F*(2, 72) = 0.52, *p* = 0.60. Introducing levels of social anxiety—assessed through levels of social worry and fear of negative evaluation—accounted for an additional 2.7% of the variation in children’s self-reported perspective-taking (FIMI), and this change in *R*^2^ was not significant, *F*(2, 70) = 0.98, *p* = 0.381. Adding levels of child-reported social avoidance accounted for an additional 5.5% of the variation in children’s self-reported perspective-taking (FIMI), and this change in *R*^2^ was significant, *F*(1, 68) = 4.18, *p* = 0.045. That is, increased self-reported avoidance behavior predicted poorer self-reported perspective-taking.

#### 3.2.5. Prosodic Emotion Recognition (CAM-C) Predicted by Social Anxiety

Hierarchical regression analyses predicting prosodic emotion recognition (Table 8) revealed that age and gender accounted for 15.8% of the variability in emotion recognition, *F*(2, 72) = 6.73, *p* = 0.002. Introducing levels of social anxiety—assessed through the cognitive measures of social worry and fear of negative evaluation—accounted for only an additional 1.5% of the variation in children’s prosodic emotion recognition, and this change in *R*^2^ was not significant, *F*(2, 70) = 0.62, *p* = 0.540. Further, adding levels of social avoidance accounted for an additional 1.7% of the variation in children’s prosodic emotion recognition, and this change in *R*^2^ was also not significant, *F*(1, 68) = 1.41, *p* = 0.239.

In summary, these analyses reveal that, when combined, our cognitive measures of social anxiety—social worry and fear of negative evaluation—did not account for a significant amount of variation in our social cognitive measures above and beyond age and gender. However, our measure of fear of negative evaluation proved to be a unique and significant predictor of certain components of social cognition. Greater child-reported fear of negative evaluation was reported as a significant predictor of poorer mental state understanding, as assessed by the CSUS parent-report. Counterintuitively, child-reported fear of negative evaluation was also a significant predictor of improved actual performance in mental state understanding tasks, as assessed by the 2-item TOM scale. Adding levels of social avoidance did not account for a significant amount of variation in prosodic emotion recognition or mental state understanding. However, social avoidance emerged as a significant predictor of additional variance in children’s self-reported social perspective-taking, with higher social avoidance associated with lower self-reported social perspective-taking. See Table 9 for an overview of the main results from the regression analyses.

### 3.3. Exploratory Analyses

Exploratory analyses were conducted exploring the same hierarchical regressions among younger and older subgroups of our original sample (using the median split technique, median age = 105 months), which may be found in Supplementary Materials—Section S3).

## 4. Discussion

The findings of the present study support the overarching hypotheses that the nature, and even direction, of the relationships between social cognition and anxiety, depend on the measure of social cognition and social anxiety under investigation. However, results were only partially consistent with our measure-specific hypotheses. When combined, our cognitive measures of social anxiety (i.e., social worry and fear of negative evaluation) did not significantly predict any of the social cognitive measures. Yet, on its own, fear of negative evaluation predicted lower scores on the parent-report measure of mental state understanding but greater accuracy in a behavioral task of mental state understanding. Adding social avoidance to these models did not explain additional variability above and beyond age, gender, and the cognitive measures of social anxiety. However, when predicting a child’s self-reported social perspective-taking, higher levels of social avoidance significantly predicted lower scores in social perspective-taking. That is, children who were more likely to report that they avoided specific social situations altogether also tended to report that they were not very good at perspective-taking (e.g., do not *‘find it easy to guess what someone else is feeling or thinking’*). Our measure of prosodic emotion recognition was not significantly predicted by any of our measured aspects of social anxiety after controlling for age and gender. Additionally, the four measures of social cognition were not significantly correlated.

The finding that fear of negative evaluation appears to play an important role in the relationship between social anxiety and social cognition is consistent with previous research, highlighting fear of negative evaluation as a key cognitive feature of social anxiety. It is also consistent with the work showing its important role in both maintaining Social Anxiety Disorder and mediating the relationship between social anxiety and other cognitive outcomes (Briscoe et al., 2024; Fredrick et al., 2020; Kocovski & Endler, 2000; Morrison et al., 2024; Sigurvinsdottir et al., 2021). In the current work, we found significant relationships between fear of negative evaluation and mental state understanding, but in opposite directions, depending on the measure. This differential pattern presents an interesting contradiction (or an apparent one). It may be that fear of negative evaluation increases a child’s vigilance or effort, and resulting accuracy, in behavioral measures of social cognition, aligning with research suggesting that social anxiety increases one’s awareness and sensitivity to social cues (Ale et al., 2010, Arrais et al., 2010; Joormann & Gotlib, 2006; Pequet & Warnell, 2021; Tibi-Elhanany & Shamay-Tsoory, 2011; Yuan et al., 2025a). At the same time, fear of negative evaluation may decrease children’s motivation, or propensity, to engage in mental state understanding (and frequency of doing so) in their daily lives for fear of what others may be thinking. In other words, while socially anxious children may have the *tools* to accurately engage in mental state understanding when asked directly, they may be less inclined to *use them* in natural settings, thus engaging in mental state understanding less frequently. These findings align with studies suggesting an important distinction between state- and trait-like mentalizing, wherein socially anxious individuals struggle to reason about the mental state of others in social settings which they find stressful (i.e., state-like) (Chevalier et al., 2023). Yet they may perform accurately in certain environments, such as a research lab where the social stimuli are more constrained, controlled, or simplistic, or where they are interacting with a single attentive and encouraging researcher. Notably, our community sample was neither selected, nor excluded, based on a clinical diagnosis of social anxiety. Although clinical status was not assessed in our sample, its prevalence likely approximates the global estimate of approximately 5% (Salari et al., 2024). Accordingly, it is important to recognize that the relationships observed in our sample may differ in a clinical population. It is also important to note that parents can be limited in their objectivity and observations of children’s engagement in mental state reasonings, particularly when they are in school and interacting with their peers. Thus, parent-reports may not fully capture children’s understanding, and may therefore differ from measures, such as structured observation by a trained researcher.

It is also worth acknowledging that these measures of mental state understanding differ not only in their objective versus subjective nature, but also in breadth. That is, the behavioral measure of mental state accuracy specifically addressed children’s understanding of only two mental states: False Beliefs and Hidden Emotions. In contrast, the other measure asked parents to reflect on their child’s observable engagement with a more diverse array of mental states that included beliefs and emotions as well as intentions, desires, preferences, and knowledge states. Moreover, the parent measure assesses children’s behavior over the course of recent weeks and across multiple social situations. As such, it is unclear whether these conflicting relationships emerged because of differences in measurement breadth, objectivity, or both. To help clarify the specific relationships between fear of negative evaluation and social cognitive processes, future work should consider replicating and expanding upon this work using a broader accuracy measure (e.g., Happe’s Strange Stories; Happé, 1995) and another subjective measure of mental state understanding (e.g., the Interpersonal Reactivity Index; Davis, 1980).

The conflicting relationships in our results, and in the broader literature, may also be partially explained by the use of different informants. We found only moderate or ‘close-to-moderate’ correlations between child- and parent-report on the anxiety measures. That these correlations were not larger, coupled with a negligible association between parent- and child-reports of perspective-taking, raises the question of the source of these discrepancies. Do the lack of correlations reflect a child’s limitations in reporting on their own thoughts and behaviors, the parent’s limitations in accurately judging their child’s social cognitive skills and feelings of anxiety, or both?

On the one hand, parents rated girls as having stronger mental state understanding than boys, yet no relationship emerged between gender and the children’s performance on either of our two behavioral measures of mental state understanding. It is thus possible that parents are simply biased in believing that their girls are more sensitive to the emotions and perspectives of others than are their boys. Notably, children’s self-reports of their own social perspective-taking also did not reflect any gender differences, consistent with our behavioral measures but not the parent-report. Similarly, the parent-report of children’s mental state understanding was not significantly correlated with age, which is inconsistent with the age-related changes observed in our behavioral measures. In contrast, children’s self-reports on social avoidance did predict their self-reported social perspective-taking. This lends further credence to arguments that children’s reports might be more accurate than parent reports in this age range (Caqueo-Urízar et al., 2022; Kiel et al., 2015; Varni et al., 2007).

On the other hand, the parent-report measure assessing how often a child reasons or speaks about the minds of others revealed a negative relationship with child-reported fear of negative evaluation. This aligns with prior research in which the CSUS positively predicted social skills, and negatively predicted social-emotional difficulties, in children (Haddock & Birch, 2024). This relationship was somewhat attenuated in samples ranging in age from 3 to 12 years compared to samples with a narrower and younger age range (ranging from 3 to 7 years), raising the possibility that the CSUS’s ability to capture individual differences in older children may be more limited (Haddock & Birch, 2024). Again, these measurement differences underscore the challenges of relying on one measure, or one informant-type, when assessing social cognition. There are undoubtedly pros and cons to both informant types. Further research is needed to determine whether one type, child- or parent-report, shows greater reliability and accuracy in assessing certain aspects of social anxiety and social cognition, or whether the two complement each other in important ways.

According to our findings, age was significantly and positively correlated with greater accuracy in prosodic emotion recognition and the 2-item TOM scale. This aligns with prior research examining the development of social cognition and its substantial improvement through early childhood (Wellman, 2014). These associations lend credence to the validity of these measures by indicating that these tasks capture the expected age-related changes in social cognitive accuracy. To our knowledge, no one has previously used a reduced-item version of the CAM-C. Thus, an additional contribution of this work might be the utility of this abbreviated measure to capture *age-related* changes in prosodic emotion detection, while minimizing the fatigue and attentional concerns posed by the full 54 item version (Golan et al., 2015). However, these findings must also be considered against measurement limitations. First, this abbreviated version does not seem ideal as an individual difference measure of prosodic emotion recognition as its internal consistency was low. Similarly, while the TOM scale is well-established in the literature as an assessment of mental state understanding, it was designed to capture age-related changes in social cognitive development and might be limited in capturing individual differences. Another limitation in the emotion recognition task was the unfamiliar accent in the voice recordings. The measure was designed and validated in the UK; therefore, the recordings used British English actors, and some of the children in our study (located in Canada) may have struggled to recognize the emotions being expressed from the voice recordings because of the unfamiliar accents. Given these limitations we remain agnostic about how to interpret the emotion recognition results and recommend further research to examine the relationships between emotion recognition and aspects of social anxiety using a variety of measures.

The limitations in the CAM-C could help to explain why the current study found no association between prosodic emotion recognition and different aspects of social anxiety. These findings did not align with our expectations, nor with prior studies specifically examining prosodic emotion recognition in children and adults (using the Diagnostic Analysis of Nonverbal Accuracy Tests), that reported poorer accuracy and slower reaction times in socially anxious individuals (McClure & Nowicki, 2001; Tseng et al., 2017). It is also possible that more interesting differences in our measures of emotion recognition and social avoidance contributed to these inconsistencies. For instance, McClure and Nowicki (2001) assessed recognition of simple emotions such as *anger*, *fear*, *sadness*, and *happiness*, whereas our measure included only complex emotions such as *unfriendly*, *jealous*, and *amused*. Additionally, their anxiety measures distinguished between children’s social avoidance in general versus novel social situations, and their findings were specific to children who showed higher levels of *general* social avoidance. Our measure did not distinguish between avoidance of general versus novel situations possibly limiting its specificity.

The lack of correlations across the four social cognition measures appears to underscore the idea that the concept of social cognition is incredibly broad and cannot be adequately captured using one measure. Thus, it would be unsurprising that associations between social cognition and other constructs (e.g., social anxiety) in previous studies, and our own, varied. However, some measures in our study showed low reliability in our sample, specifically the measures of prosodic emotion recognition, self-reported social perspective-taking, and social avoidance. Poor reliability in measures can indicate that the items failed to capture the same construct (Menon et al., 2025), which could explain the lack of correlations between our measures of social cognition. Given these measurement limitations, our results using those measures should be interpreted with caution. Unfortunately, these measurement challenges are not unique to our study and reflect a broader issue in the field of social cognitive development that needs to be addressed in future research. In comparison to these measures of potential concern, the parent-report measure of mental state understanding (Tahiroglu et al., 2014) showed excellent reliability and previous work has shown its predictive validity with several facets of children’s social emotional health (e.g., Haddock & Birch, 2024). As such, we are most confident in the finding that higher levels of the fear of negative evaluation are associated with lower engagement in children’s mental state reasoning in their every daily lives.

We were sufficiently powered to detect medium and large effects; nonetheless future research would benefit from larger sample sizes to increase sensitivity to detect small effects and conduct Structural Equation Modeling to minimize the effects of measurement error and low reliability. None of the effect sizes observed were particularly large and a substantial proportion of the variance went unaccounted for in our dependent variables, even for significant effects. Future research might benefit from including other theoretically relevant variables such as children’s language comprehension and executive function. Finally, although our procedure minimized fatigue effects (e.g., ensuring participants wished to continue), the measure order could have introduced systematic variance. Our measures were presented in the same order, with those that are more vulnerable to fatigue, such as the prosodic emotion recognition task, completed earliest. Future research could consider varying the order of the tasks.

## 5. Conclusions

In sum, our findings suggest that social cognition is a broad construct encompassing multiple cognitive processes that have varied associations with other constructs, such as different aspects of social anxiety. We found that greater fear of negative evaluation was a significant predictor of increased accuracy in mental state understanding, at least in terms of reasoning about False Beliefs and Hidden Emotions. In comparison, greater fear of negative evaluation was a significant predictor of lower parent-reported mental state understanding in their child’s day-to-day behavior. In addition, social avoidance accounted for additional variance in children’s self-reported social perspective-taking.

These findings highlight the importance of distinguishing between mental state understanding (i.e., the capacity to reason accurately about others’ mental states) and the propensity to do so (i.e., the ease and frequency with which one thinks about others’ mental states). Although these often align, they are distinct, as illustrated by the earlier examples of over- and under-mentalizing. Unfortunately, many existing measures of social cognition conflict between these components. A clearer distinction between children’s competence and propensity may be especially useful for predicting their social skills and social-emotional well-being.

Together, our results suggest that understanding social cognitive ability and its relationship to social anxiety requires a multifaceted, multi-measure approach. We therefore encourage researchers to specify the constructs and measures involved when summarizing findings on broad concepts such as social anxiety and social cognition. As noted above, some of our findings should be interpreted cautiously because some measures showed low reliability. Replications and extensions using additional measures are needed to validate our findings and interpretations. Finally, while our results show that associations between social cognition and social anxiety are measure-dependent, we cannot determine how much of this variation reflects differences in the components examined versus differences in measurement type or informant. Future research should prioritize disentangling these measurement differences while developing and validating stronger assessment tools.

This research is particularly relevant today, as a generation of young children experienced a substantial reduction in social interactions due to the COVID-19 pandemic, which negatively impacted social cognitive development (Alan & Turkum, 2024; Rodriguez-Monge et al., 2023) and increased levels of social anxiety (Peçanha et al., 2025). Understanding the factors that reduce anxiety and support children’s capacity and motivation to engage in social cognition is critical for fostering their social-emotional wellbeing.

## Figures and Tables

**Table 1:** Description of measures and example items.

Measure	Description and Example Items
Cambridge Mindreading Face-Voice Battery for Children (CAM-C) (Golan et al., 2015)	10-item battery measuring children’s accuracy in prosodic emotion recognition. Respondents listen to a voice recording and are presented with four options, scoring 1 point for each correct response. Minimum score = 0 and Maximum score = 10. Sums of scores used in analysis Voice recording: “I wish I didn’t have to do it.” 1. Disbelieving; 2. Bothered (correct); 3. Liked; 4. Impressed
Theory of Mind Scale (TOM scale; Wellman & Liu, 2004) Child behavioral task	Two-task battery measuring children’s mental state understanding, including False Beliefs and Hidden Emotions. Minimum score = 0 and Maximum score = 2. Higher scores indicated greater mental state understanding, and sums of scores were used in analysis. False Belief “Peter has never ever seen inside this Band-Aid box [that child knows contains a car]. What does Peter think is in the box?” 1—Band-Aids (correct); 2—Toy Car Hidden Emotions “How did Matt really feel, when everyone laughed?” 1—Happy; 2—Okay; 3—Sad “How did Matt try to look on his face?” 1—Happy; 2—Okay; 3—Sad
Children’s Social Understanding Scale (CSUS; Tahiroglu et al., 2014) Parent report	18-item parent-report measure assessing their child’s understanding of mental states with answers ranging from 1 to 4. Minimum score = 4 and Maximum score = 72. Higher scores indicate greater mental state understanding, and sums of scores were used in analysis Example item: *My child talks about differences in what people like or want (e.g., “you like coffee, but I like juice”)*
Four Item Mentalizing Index (FIMI; Clutterbuck et al., 2021)	4-item child- and parent-report measures assessing their tendency to engage in social perspective-taking with answers ranging from 1 to 4. Minimum score = 4 and Maximum score = 16. Higher scores indicate greater self-reported perspective-taking, and sums of scores were used in analysis. Example item: *I/My child sometimes try/tries to understand my/their friends better by imagining what they are thinking or feeling.*
Social Worries Anxiety Index for Young Children (SWAIY; Spence, 1995); modified into SWAIY-M and SWAIY-A	12-item child- and parent-report measure assessing levels of social worry and social avoidance. Social worry answers ranged from 0 to 2. Minimum score = 0 and Maximum score = 24. Social avoidance answers were coded as a binary ‘yes’ or ‘no’ response. Minimum score = 0 and Maximum score = 24. Higher scores indicate greater levels of social worry and avoidance, and sums of scores were used in analysis. Example items: *I/My child get/s worried about going to parties or playdates.*
Brief Fear of Negative Evaluation (BFNE; Leary, 1983)	8-item child-report measure assessing their fear of negative evaluation with answers ranging from 1 to 5. Minimum score = 8 and Maximum score = 40. Higher scores indicated increased fear of negative evaluation, and sums of scores were used in analysis. Example item: *I/My child am/is afraid that other people will notice my/their mistakes.*

**Table 2:** Descriptive statistics for age in months, gender, social anxiety and social cognitive measures—child-report (CR) and parent-report (PR).

Variable	*M*	*SD*	Min	Max	*N*
Age—months (years)	103.65 (8.15)	19.58 (1.61)	72 (6)	155 (12)	78
CAM-C	6.49	1.98	2	10	78
TOM scale	0.83	0.30	0	1.00	78
CSUS (PR)	61.43	7.57	41	72	74
SWAIY-M (CR)	8.12	4.33	0	18	77
SWAIY-A (CR)	1.51	1.47	0	6	76
BFNE (CR)	17.49	7.16	8	38	78
FIMI (CR)	10.83	2.17	5	16	78

**Table 3:** Zero-order correlations for age in months, gender, social anxiety and social cognitive measures.

Variable		1	2	3	4	5	6	7	8	9
1. Age months		-								
2. Gender	*r*	−0.08								
	*p*	0.51	-							
	*N*	76								
3. CAM-C	*r*	0.38 **	0.10							
	*p*	<0.001	0.402	-						
	*N*	78	76							
4. TOM	*r*	0.25 *	−0.18	0.13						
	*p*	0.026	0.118	0.245	-					
	*N*	78	76	78						
5. CSUS (PR)	*r*	−0.14	−0.05	−0.05	0.01					
	*p*	0.247	0.661	0.704	0.955	-				
	*N*	74	74	74	74					
6. FIMI (CR)	*r*	0.13	−0.01	0.08	0.05	0.03				
	*p*	0.260	0.930	0.472	0.696	−0.784	-			
	*N*	78	72	78	78	74				
7. SWAIY-M (CR)	*r*	−0.02	0.06	−0.07	−0.05	−0.04	−0.06			
	*p*	0.886	0.587	0.540	0.668	0.731	0.593	-		
	*N*	77	75	77	77	73	77			
8. SWAIY-A (CR)	*r*	0.12	0.03	−0.08	0.05	0.01	−0.22	0.45 **		
	*p*	0.308	0.825	0.472	0.666	0.934	0.059	<0.001	-	
	*N*	76	74	76	76	72	76	76		
9. BFNE (CR)	*r*	0.24 *	0.08	0.14	0.19	−0.26 *	−0.04	0.56 **	0.41 **	
	*p*	0.032	0.486	0.222	0.093	0.025	0.740	<0.001	<0.001	-
	*N*	78	76	78	78	74	78	77	76	

* *p* < 0.05, ** *p* < 0.01.

**Table 4:** Correlations for social cognitive components—controlling for age.

Variable		CAM-C	TOM	CSUS	FIMI (CR)
1. CAM-C	*r*				
	*p*	-			
	*N*				
2. TOM	*r*	0.04			
	*p*	0.716	-		
	*N*	75			
3. CSUS	*r*	0.01	0.04		
	*p*	0.751	0.719	-	
	*N*	75	71		
4. FIMI (CR)	*r*	0.04	0.01	0.05	
	*p*	0.751	0.911	0.669	-
	*N*	75	75	71	

**Table 5:** Summary of Hierarchical Regression Analysis for gender, age, and social anxiety measures predicting mental state understanding (CSUS).

Predictor Variables		*B*	β	*t*	*R* ^2^	Δ*R*^2^
Model 1					0.02	0.022
	Age in months	−0.007	−0.14	−1.19		
	Gender of child	−0.12	−0.06	−0.49		
Model 2					0.09	0.067
	Age in months	−0.003	−0.06	−0.26		
	Gender of child	−0.06	−0.03	−0.26		
	Social worry (SWAIY-M)	0.14	0.14	0.98		
	Fear of negative evaluation (BFNE)	−0.33	−0.32	−2.21 *		
Model 3					0.09	0.008
	Age in months	−0.004	−0.09	−0.68		
	Gender of child	−0.05	−0.02	−0.21		
	Social worry (SWAIY-M)	0.09	0.09	0.62		
	Fear of negative evaluation (BFNE)	−0.32	−0.31	−2.05 *		
	Social Avoidance (SWAIY-A)	0.07	0.10	0.77		

* *p* < 0.05.

**Table 6:** Summary of Hierarchical Regression Analysis for gender, age, and social anxiety measures predicting mental state understanding (2-item TOM scale).

Predictor Variables		*B*	β	*t*	*R* ^2^	Δ*R*^2^
Model 1					0.10	0.097 *
	Age in months	0.01	0.25	2.21 *		
	Gender of child	−0.36	−0.17	−1.52		
Model 2					0.15	0.050
	Age in months	0.009	0.18	1.53		
	Gender of child	−0.40	−0.19	−1.69		
	Social worry (SWAIY-M)	−0.21	−0.21	−1.55		
	Fear of negative evaluation (BFNE)	0.28	0.72	1.96		
Model 3					0.15	0.000
	Age in months	0.008	0.16	1.31		
	Gender of child	−0.38	−0.18	−1.61		
	Social worry (SWAIY-M)	−0.23	−0.23	−1.59		
	Fear of negative evaluation (BFNE)	0.32	0.31	2.17 *		
	Social Avoidance (SWAIY-A)	−0.01	−0.01	−0.09		

* *p* < 0.05.

**Table 7:** Summary of Hierarchical Regression Analysis for gender, age, and child-reported social anxiety measures predicting a child’s self-reported social perspective-taking (FIMI).

Predictor Variables		*B*	β	*t*	*R* ^2^	Δ*R*^2^
Model 1					0.01	0.014
	Age in months	0.006	0.02	0.17		
	Gender of child	0.007	0.04	0.30		
Model 2					0.04	0.027
	Age in months	0.008	0.16	1.30		
	Gender of child	0.08	0.04	0.33		
	Social worry (SWAIY-M)	−0.008	−0.008	−0.05		
	Fear of negative evaluation (BFNE)	−0.16	−0.17	−1.12		
Model 3					0.11	0.055 *
	Age in months	0.01	0.21	1.71		
	Gender of child	0.06	0.03	0.25		
	Social worry (SWAIY-M)	0.10	0.10	0.71		
	Fear of negative evaluation (BFNE)	−0.16	−0.16	−1.08		
	Social Avoidance (SWAIY-A)	−0.18	−0.27	−2.05 *		

* *p* < 0.05.

**Table 8:** Summary of Hierarchical Regression Analysis for gender, age, and social anxiety predicting prosodic emotion recognition (CAM-C).

Predictor Variables		*B*	β	*t*	*R* ^2^	Δ*R*^2^
Model 1					0.16	0.158 **
	Age in months	0.02	0.39	3.55 **		
	Gender of child	0.27	0.13	1.17		
Model 2					0.17	0.015
	Age in months	0.02	0.35	3.08 **		
	Gender of child	0.26	0.12	1.11		
	Social worry (SWAIY-M)	−0.14	−0.14	−1.06		
	Fear of negative evaluation (BFNE)	0.13	0.12	0.88		
Model 3					0.19	0.017
	Age in months	0.02	0.38	3.27 **		
	Gender of child	0.25	0.12	1.05		
	Social worry (SWAIY-M)	−0.08	−0.08	−0.56		
	Fear of negative evaluation (BFNE)	0.13	0.12	0.85		
	Social Avoidance (SWAIY-A)	−0.15	−0.15	−1.19		

** *p* < 0.01.

**Table 9:** Overview of the main results.

	Prosodic Emotion Recognition (CAM-C—Behavioral Task	Mental State Understanding (TOM Scale— Behavioral Task)	Mental State Understanding (CSUS— Parent-Report)	Social Perspective-Taking (FIMI—Child-Report)
Fear of Negative Evaluation—child-report	-	FNE predicts greater accuracy in TOM	FNE predicts lower parent-report ratings on CSUS	-
Social Avoidance—child-report	-	-	-	Social avoidance predicts a lower tendency

## Data Availability

The data that support the findings of this study are openly available in Open Science Framework (OSF) at https://osf.io/87ew5 (assess on 27 October 2025).

## Supplementary Materials

The following supporting information can be downloaded at: https://www.mdpi.com/article/10.3390/bs16071228/s1, Section S1: Full details of all items and measures; Section S2: Parent-report Regressions; Section S3: Exploratory analyses by age (median split); Section S4: Variance Inflation Factor values.
