# Supplementary material for: The Anxious Mind: Examining the Conflicting Relationships Between Distinct Aspects of Social Anxiety and Social Cognition
Source: Behav Sci (Basel). 2026 Jul 19;16(7):1228. doi: 10.3390/bs16071228 (PMC13405778; doi:10.3390/bs16071228)
Supplement: Supplementary file 1 [file behavsci-16-01228-s001.zip › behavsci-4364921-supplementary.pdf]

**Supplemental Materials for Anxious Mind: Examining the conflicting relationships between distinct aspects of social anxiety and social cognition**

**Supplementary Section S1:** ***Full details of all items and measures***

**SWAIY-M**

Instructions: Now I'm going to read you some sentences to you, and I want to know how much they sound like you!

For example, if I say "I like bunnies",

If you don't like bunnies at all, you say 0, NOT AT ALL like me, but if you like them a little bit, you say, 1, A LITTLE like me , or if you like them a lot, you say 2, A LOT like me.

Let's start with the first sentence, "I get worried about going to parties or playdates". Worried means to feel a bit nervous about something.

Does that sound not at all like you? A little like you? Or a lot like you?

1. I get worried about going to parties or play dates.
2. I get worried about speaking on the telephone.
3. I get worried about meeting new people.
4. I get worried about presenting work to the class, putting up my hand, or speaking in front of the class (show & tell).
5. I get worried about attending groups, clubs, or after school activities.
6. I get worried about approaching groups of kids to ask to join in (or play).
7. I get worried about talking in front of a group of adults.
8. I get worried about going into a shop alone or telling staff in a cafe what I would like.
9. I get worried about standing up for myself with other kids i.e. when someone takes my toy.
10. I get worried about entering a room full of people.
11. I get worried about using public toilets.
12. I get worried about eating in public.

**SWAIY-avoidance**

Instructions: Next, think about the situation... would it make you so nervous or worried that you would avoid going to the playdate? or would you still go anyway? Avoid means to stay away from someone or something.

Pick "Yes" if you would avoid it or "No" if you would still go.

1. Would you get so worried about going to parties or play dates that you would avoid it completely?
2. Would you get so worried about speaking on the telephone that you would avoid it completely?
3. Would you get so worried about meeting new people that you would avoid it completely?
4. Would you get so worried about presenting work to the class, putting up my hand, or speaking in front of the class (show & tell) that you would avoid it completely?
5. Would you get so worried about attending groups, clubs, or after school activities that you would avoid it completely?
6. Would you get so worried about approaching groups of kids to ask to join in (or play) that you would avoid it completely?
7. Would you get so worried about talking in front of a group of adults that you would avoid it completely?
8. Would you get so worried about going into a shop alone or telling staff in a cafe what I would like that you would avoid it completely?
9. Would you get so worried about standing up for myself with other kids i.e. when someone takes my toy that you would avoid it completely?
10. Would you get so worried about entering a room full of people that you would avoid it completely?
11. Would you get so worried about using public toilets that you would avoid it completely?
12. Would you get so worried about eating in public that you would avoid it completely?

**Four Item Mentalizing Index – child version**

Instructions: Now I am going to read you a series of sentences about yourself.

After I say each sentence, I want you to tell me if it's:

- Definitely not like you 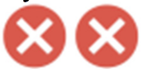
- Not like you 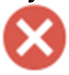
- A little like you 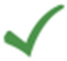
- A lot like you 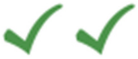

Does that make sense? Let's give it a try!

1. I find it easy to tell how others are feeling.

2. I find it hard to guess what other people are thinking or feeling.
3. I sometimes try to understand my friends better by imagining what they are thinking or feeling.
4. I can usually understand what others think, even if it's not the same as what I think.

**Supplementary Section S2:** ***Parent-report Regressions***

**Parent Report Regressions**

***Prosodic emotion recognition (CAM-C).***

Hierarchical regression analyses predicting prosodic emotion recognition (Table S1) revealed that age and gender accounted for approximately 14.9% of its variability, $F(2, 71) = 6.24, p = .003$. Introducing levels of social anxiety – assessed through levels of social worry and fear of negative evaluation – accounted for only an additional 2.5% of the variation in children's prosodic emotion recognition, and this change in $R^2$ was not significant, $F(2, 69) = 1.04, p = .360$. Adding levels of social avoidance accounted for zero additional variation in children's prosodic emotion recognition, and this change in $R^2$ was not significant, $F(1, 51) = .002, p = .966$.

**Table S1**

*Summary of Hierarchical Regression Analysis for gender, age, and social anxiety predicting prosodic emotion recognition (CAM-C).*

| Predictor Variables | | *B* | β | *t* | $R^2$ | $\Delta R^2$ |
|---|---|---|---|---|---|---|
| Model 1 | | | | | .15 | **.149**** |
| | Age in months | .02 | .36 | **3.30**** | | |
| | Gender of child | .33 | .16 | 1.46 | | |
| Model 2 | | | | | .17 | .025 |
| | Age in months | .02 | .40 | **3.47**** | | |

| | | | | | | | |
|---|---|---|---|---|---|---|---|
| | Gender of child | .34 | | .17 | 1.53 | | |
| | Social worry (SWAIY-M) | -.05 | | -.05 | -.43 | | |
| | Fear of negative evaluation (BFNE) | -.13 | | -.13 | -1.12 | | |
| Model 3 | | | | | | .21 | .00 |
| | Age in months | .02 | | .39 | **3.11**** | | |
| | Gender of child | .46 | | .22 | 1.77 | | |
| | Social worry (SWAIY-M) | -.15 | | -.15 | -1.00 | | |
| | Fear of negative evaluation (BFNE) | -.09 | | - .098 | -.68 | | |
| | Social Avoidance (SWAIY-A) | .006 | | .006 | .04 | | |

*$p$ < .05, **$p$ < .01

***Mental state understanding (TOM scale).***

Hierarchical regression analyses predicting mental state understanding as assessed by the TOM scale (Table S2) revealed that age and gender accounted for approximately 10.6% of its variability, $F(2, 71) = 4.22$, $p = .019$. Introducing levels of social anxiety – assessed through levels of social worry and fear of negative evaluation – accounted for only an additional 9.5% of the variation in children’s mental state understanding (TOM scale), and this change in $R^2$ was not significant, $F(2, 69) = 1.54$, $p = .223$. Adding levels of social avoidance accounted for an

additional 0.2% of the variation in children's mental state understanding (TOM scale), and this change in $R^2$ was not significant, $F(1, 51) = .146, p = .704$.

**Table S2**

*Summary of Hierarchical Regression Analysis for gender, age, and social anxiety measures predicting mental state understanding (TOM scale)***.**

| Predictor Variables | | *B* | β | *t* | $R^2$ | $\Delta R^2$ |
|---|---|---|---|---|---|---|
| Model 1 | | | | | .11 | **.106*** |
| | Age in months | .01 | .26 | **2.33*** | | |
| | Gender of child | -.24 | -.097 | -.59 | | |
| Model 2 | | | | | .14 | .038 |
| | Age in months | .02 | .31 | **2.71**** | | |
| | Gender of child | -.36 | -.17 | -1.54 | | |
| | Social worry (SWAIY-M) | -.21 | -.21 | -1.70 | | |
| | Fear of negative evaluation (BFNE) | .03 | .03 | .23 | | |
| Model 3 | | | | | .20 | .002 |
| | Age in months | .02 | .36 | **2.83**** | | |
| | Gender of child | -.49 | -.22 | -1.75 | | |
| | Social worry (SWAIY-M) | -.18 | -.16 | -1.12 | | |
| | Fear of negative evaluation (BFNE) | .06 | .06 | .37 | | |
| | Social Avoidance (SWAIY-A) | -.06 | -.06 | -.38 | | |

*$p < .05$, **$p < .01$

***Mental state understanding (CSUS).***

Hierarchical regression analyses predicting mental state understanding as assessed by the CSUS parent-report (Table S3) revealed that age and gender accounted for approximately 2.2% of its variability, $F(2, 71) = .80$, $p = .45$. Introducing levels of social anxiety – assessed through levels of social worry and fear of negative evaluation – accounted for an additional 3.8% of the variation in children's mental state understanding (CSUS), and this change in $R^2$ was not significant, $F(2, 69) = 1.38$, $p = .257$. Adding levels of social avoidance accounted for only an additional 2.1% of children's mental state understanding (CSUS), and this change in $R^2$ was not significant, $F(1, 51) = 1.22$, $p = .275$.

Furthermore, fear of negative evaluation (BFNE) emerged as a unique and significant predictor of mental state understanding as assessed by the CSUS parent-report measure. A higher fear of negative evaluation was associated with lower ratings of mental state understanding, $t(51) = -2.31$, $p = .025$.

**Table S3**

*Summary of Hierarchical Regression Analysis for gender, age, and social anxiety measures predicting mental state understanding (CSUS).*

| Predictor Variables | | *B* | β | *t* | $R^2$ | $\Delta R^2$ |
|---|---|---|---|---|---|---|
| Model 1 | | | | | .02 | .022 |
| | Age in months | -.007 | -.14 | -1.19 | | |
| | Gender of child | -.13 | -.06 | -.51 | | |
| Model 2 | | | | | .06 | .038 |
| | Age in months | -.005 | -.10 | -.84 | | |
| | Gender of child | -.11 | -.05 | -.43 | | |

| | | | | | | |
|---|---|---|---|---|---|---|
| | Social worry (SWAIY-M) | -.04 | -.04 | -.33 | | |
| | Fear of negative evaluation (BFNE) | -.18 | -.18 | -1.40 | | |
| Model 3 | | | | | .13 | .021 |
| | Age in months | -.005 | -.098 | -.74 | | |
| | Gender of child | -.08 | -.04 | -.29 | | |
| | Social worry (SWAIY-M) | -.07 | -.07 | -.43 | | |
| | Fear of negative evaluation (BFNE) | -.33 | -.35 | **-2.31**** | | |
| | Social Avoidance (SWAIY-A) | .17 | .17 | 1.10 | | |

*$p$ < .05, **$p$ < .01

***Social perspective taking (FIMI).***

Hierarchical regression analyses predicting child-reported social perspective taking (Table S4) revealed that age and gender accounted for 5.7% of its variability, $F(2, 69) = .2.08$, $p = .13$. However, gender emerged as a unique and significant predictor of children's self-reported perspective taking (FIMI), with girls receiving higher scores, $t(50) = 2.096$, $p = .041$.

Introducing levels of social anxiety – assessed through levels of social worry and fear of negative evaluation – accounted for an additional 1.8% of the variation in children's self-reported perspective taking (FIMI), and this change in $R^2$ was not significant, $F(2, 67) = .598$, $p = .553$. Adding levels of social avoidance accounted for an additional 0.1% of the variation in

children's self-reported perspective taking (FIMI), and this change in $R^2$ was not significant, $F(1, 50) = .040$, $p = .842$.

**Table S4**

*Summary of Hierarchical Regression Analysis for gender, age, and social anxiety measures predicting a child's parent-reported social perspective taking (FIMI).*

| Predictor Variables | | *B* | β | *t* | $R^2$ | $\Delta R^2$ |
|---|---|---|---|---|---|---|
| Model 1 | | | | | .06 | .057 |
| | Age in months | .001 | .01 | .09 | | |
| | Gender of child | .498 | .24 | **2.04*** | | |
| Model 2 | | | | | .07 | .017 |
| | Age in months | .002 | .05 | .38 | | |
| | Gender of child | .50 | .24 | **2.05*** | | |
| | Social worry (SWAIY-M) | -.13 | -.13 | -1.02 | | |
| | Fear of negative evaluation (BFNE) | -.001 | -.001 | -.01 | | |
| Model 3 | | | | | .30 | .003 |
| | Age in months | .003 | .07 | .47 | | |
| | Gender of child | .63 | .29 | **2.096*** | | |
| | Social worry (SWAIY-M) | -.16 | -.15 | -.97 | | |
| | Fear of negative evaluation (BFNE) | -.03 | -.03 | -.16 | | |

| | | | |
|---|---|---|---|
| Social Avoidance (SWAIY-A) | -.04 | -.03 | -.20 |

*$p < .05$, **$p < .01$

**Discussion**

In out parent-report regressions, we found that children's fear of negative evaluation (as reported by their parents) was still a significant and negative predictor of their mental state understanding in their daily lives, $t(51) = -2.31$, $p = .025$, ($p = .13$ after False Discovery Rate correction). However, in contrast to our child-report regressions, fear of negative evaluation did not emerge as a significant predictor of children's accuracy in mental state understanding as assessed by the 2-item TOM scale, $t(51) = .373$, $p = .71$. These findings support the overall idea that social cognition is a broad concept, encompassing multiple cognitive processes that cannot be captured in one measure or by one informant-type. It also suggests that there may be a key distinction between what children *can* do in an artificial lab setting and how they *actually* behave in their daily lives and where their parents can observe their behaviour. Moreover, it indicates that children's perceptions of their levels of fear of negative evaluation differ from their parents'. Indeed, child- and parent-reports only approached a moderate correlation ($r = .28$, $p = .02$).

Finally, gender significantly predicted parents' ratings of their children's willingness and capacity to engage in social perspective taking, with girls rated higher than boys, $t(50) = 2.096$, $p = .04$, ($p = .21$ after False Discovery Rate correction). These findings suggest that parents are biased toward perceiving girls as more likely to take the perspectives of others, as children themselves did not show or report any gender differences in either mentalizing or social anxiety.

**Supplementary Section S3:** ***Exploratory Analyses by age (median split)***

**Young subgroup (< 8 years old)**

***Prosodic emotion recognition (CAM-C).***

Hierarchical regression analyses predicting prosodic emotion recognition (Table S5) revealed that age and gender accounted for approximately 4.0% of its variability, $F(2, 35) = .758$, $p = .476$. Introducing levels of social anxiety – assessed through levels of social worry and fear of negative evaluation – accounted for only an additional 1.9% of the variation in children's prosodic emotion recognition, and this change in $R^2$ was not significant, $F(2, 34) = .347$, $p = .709$. Adding levels of social avoidance accounted for an additional 0.2% of the variation in children's prosodic emotion recognition, and this change in $R^2$ was not significant $F(1, 32) = .065$, $p = .800$.

**Table S5**

*Summary of Hierarchical Regression Analysis for gender, age, and social anxiety predicting nonverbal emotion recognition (CAM-C).*

| Predictor Variables | | *B* | β | *t* | $R^2$ | $\Delta R^2$ |
|---|---|---|---|---|---|---|
| Model 1 | | | | | .04 | .040 |
| | Age in months | .02 | .20 | 1.22 | | |
| | Gender of child | .16 | .007 | .05 | | |
| Model 2 | | | | | .06 | .019 |
| | Age in months | .02 | .20 | 1.17 | | |
| | Gender of child | .08 | .04 | .22 | | |
| | Social worry (SWAIY-M) | -.15 | -.16 | -.82 | | |

| | | | | | | | |
|---|---|---|---|---|---|---|---|
| | Fear of negative evaluation (BFNE) | .11 | | .095 | .498 | | |
| Model 3 | | | | | | .06 | .002 |
| | Age in months | .02 | | .21 | 1.18 | | |
| | Gender of child | .41 | | .02 | .11 | | |
| | Social worry (SWAIY-M) | -.10 | | -.11 | -.49 | | |
| | Fear of negative evaluation (BFNE) | .04 | | .04 | .18 | | |
| | Social Avoidance (SWAIY-A) | -.04 | | -.05 | -.26 | | |

*$p < .05$, **$p < .01$

***Mental state understanding (TOM scale).***

Hierarchical regression analyses predicting mental state understanding as assessed by the TOM scale (Table S6) revealed that age and gender accounted for approximately 4.7% of its variability, $F(2, 36) = .891, p = .419$. Introducing levels of social anxiety – assessed through levels of social worry and fear of negative evaluation – accounted for only an additional 9.2% of the variation in children's mental state understanding (TOM scale), and this change in $R^2$ was not significant, $F(2, 34) = 1.82, p = .178$. Adding levels of social avoidance accounted for zero additional variation in children's mental state understanding (TOM scale), and this change in $R^2$ was not significant, $F(1, 32) = .001, p = .978$.

However, child-reported fear of negative evaluation (BFNE) emerged as a unique and marginally significant predictor of mental state understanding as assessed by the TOM scale,

with a higher fear of negative evaluation associated with greater accuracy in mental state understanding, $t(32) = 2.03$, $p = .051$, ($p = .26$ after False Discovery Rate correction).

**Table S6**

*Summary of Hierarchical Regression Analysis for gender, age, and social anxiety measures predicting mental state understanding (TOM scale)***.**

| Predictor Variables | | *B* | β | *t* | $R^2$ | $\Delta R^2$ |
|---|---|---|---|---|---|---|
| Model 1 | | | | | .05 | .047 |
| | Age in months | .02 | .19 | 1.13 | | |
| | Gender of child | -.24 | -.097 | -.59 | | |
| Model 2 | | | | | .14 | .092 |
| | Age in months | .02 | .15 | .94 | | |
| | Gender of child | -.11 | -.04 | -.26 | | |
| | Social worry (SWAIY-M) | -.298 | -.27 | -1.49 | | |
| | Fear of negative evaluation (BFNE) | .41 | .31 | 1.72 | | |
| Model 3 | | | | | .18 | .00 |
| | Age in months | .02 | .14 | .842 | | |
| | Gender of child | -.03 | -.01 | -.06 | | |
| | Social worry (SWAIY-M) | -.34 | -.32 | -1.56 | | |
| | Fear of negative evaluation (BFNE) | .53 | .39 | 2.03 | | |
| | Social Avoidance (SWAIY-A) | .007 | .005 | .03 | | |

$*p < .05$, $**p < .01$

***Mental state understanding (CSUS).***

Hierarchical regression analyses predicting mental state understanding as assessed by the CSUS parent-report (Table S7) revealed that age and gender accounted for approximately 1.4% of its variability, $F(2, 34) = .24$, $p = .788$. Introducing levels of social anxiety – assessed through levels of social worry and fear of negative evaluation – accounted for an additional 2.4% of the variation in children's mental state understanding (CSUS), and this change in $R^2$ was significant, $F(2, 32) = 5.04$, $p = .013$. Adding levels of social avoidance accounted for only an additional 1.4% of children's mental state understanding (CSUS), and this change in $R^2$ was not significant, $F(1, 30) = .529$, $p = .472$.

Furthermore, child-reported fear of negative evaluation (BFNE) emerged as a unique and significant predictor of mental state understanding in younger children, as assessed by the CSUS parent-report measure. A higher fear of negative evaluation was associated with lower ratings of mental state understanding, $t(30) = -2.83$, $p = .008$, ($p = .04$ after False Discovery Rate correction).

**Table S7**

*Summary of Hierarchical Regression Analysis for gender, age, and social anxiety measures predicting mental state understanding (CSUS).*

| Predictor Variables | | *B* | β | *t* | $R^2$ | $\Delta R^2$ |
|---|---|---|---|---|---|---|
| Model 1 | | | | | .01 | .014 |
| | Age in months | -.006 | -.06 | -.37 | | |
| | Gender of child | -.22 | -.11 | -.61 | | |
| Model 2 | | | | | .25 | **.236*** |

| | | | | | | |
|---|---|---|---|---|---|---|
| | Age in months | .001 | .01 | .06 | | |
| | Gender of child | -.35 | -.17 | -1.09 | | |
| | Social worry (SWAIY-M) | .29 | .32 | 1.85 | | |
| | Fear of negative evaluation (BFNE) | -.59 | -.54 | **-3.12**** | | |
| Model 3 | | | | | .23 | .014 |
| | Age in months | .002 | .02 | .15 | | |
| | Gender of child | -.37 | -.18 | -1.09 | | |
| | Social worry (SWAIY-M) | .23 | .26 | 1.28 | | |
| | Fear of negative evaluation (BFNE) | -.597 | -.54 | **-2.83**** | | |
| | Social Avoidance (SWAIY-A) | .15 | .14 | .73 | | |

**p* < .05, ***p* < .01

***Social perspective taking (FIMI).***

Hierarchical regression analyses predicting child-reported social perspective taking (Table S8) revealed that age and gender accounted for 22.4% of its variability, and this change in $R^2$ was significant $F(2, 36) = 5.12$, $p = .011$. Introducing levels of social anxiety – assessed through levels of social worry and fear of negative evaluation – accounted for an additional 4.5% of the variation in children's self-reported perspective taking (FIMI), and this change in $R^2$ was not significant, $F(2, 34) = 1.05$, $p = .361$. Adding levels of social avoidance accounted for an

additional 0.3% of the variation in children's self-reported perspective taking (FIMI), and this change in $R^2$ was not significant, $F(1, 32) = .149$, $p = .702$.

**Table S8**

*Summary of Hierarchical Regression Analysis for gender, age, and social anxiety measures predicting a child's self-reported social perspective taking (FIMI).*

| Predictor Variables | | *B* | β | *t* | $R^2$ | $\Delta R^2$ |
|---|---|---|---|---|---|---|
| Model 1 | | | | | .22 | **.224*** |
| | Age in months | .05 | .47 | **3.21**** | | |
| | Gender of child | .004 | .002 | .01 | | |
| Model 2 | | | | | .27 | .045 |
| | Age in months | .06 | .52 | **3.44**** | | |
| | Gender of child | .05 | .02 | .15 | | |
| | Social worry (SWAIY-M) | -.12 | -.12 | -.73 | | |
| | Fear of negative evaluation (BFNE) | -.16 | -.14 | -.80 | | |
| Model 3 | | | | | .30 | .003 |
| | Age in months | .06 | .54 | **3.49**** | | |
| | Gender of child | -.007 | -.003 | -.02 | | |
| | Social worry (SWAIY-M) | -.06 | -.06 | -.30 | | |
| | Fear of negative evaluation (BFNE) | -.25 | -.21 | -1.17 | | |

| | | | | | | |
|---|---|---|---|---|---|---|
| | Social Avoidance (SWAIY-A) | -.08 | -.07 | -.39 | | |

*p < .05, **p < .01

**Old subgroup (> 8 years old)**

***Prosodic emotion recognition (CAM-C).***

Hierarchical regression analyses predicting prosodic emotion recognition (Table S9) revealed that age and gender accounted for approximately 12.5% of its variability, $F(2, 33) = 2.35$, $p = .111$. Introducing levels of social anxiety – assessed through levels of social worry and fear of negative evaluation – accounted for only an additional 0.3% of the variation in children's prosodic emotion recognition, and this change in $R^2$ was not significant, $F(2, 31) = .052$, $p = .950$. Adding levels of social avoidance accounted for an additional 2.9% of the variation in children's prosodic emotion recognition, and this change in $R^2$ was not significant, $F(1, 30) = 1.02$, $p = .320$.

**Table S9**

*Summary of Hierarchical Regression Analysis for gender, age, and social anxiety predicting prosodic emotion recognition (CAM-C).*

| Predictor Variables | | *B* | β | *t* | $R^2$ | $\Delta R^2$ |
|---|---|---|---|---|---|---|
| Model 1 | | | | | .13 | .125 |
| | Age in months | .009 | .11 | .64 | | |
| | Gender of child | .58 | .31 | 1.83 | | |
| Model 2 | | | | | .13 | .003 |
| | Age in months | .008 | .09 | .52 | | |

| | | | | | | |
|---|---|---|---|---|---|---|
| | Gender of child | .54 | .29 | 1.56 | | |
| | Social worry (SWAIY-M) | -.07 | - .07 | -.27 | | |
| | Fear of negative evaluation (BFNE) | .07 | .08 | .32 | | |
| Model 3 | | | | | .16 | .029 |
| | Age in months | .01 | .13 | .73 | | |
| | Gender of child | .48 | .25 | 1.35 | | |
| | Social worry (SWAIY-M) | -.03 | - .04 | -.14 | | |
| | Fear of negative evaluation (BFNE) | .12 | .14 | .52 | | |
| | Social Avoidance (SWAIY-A) | -.17 | - .19 | -1.01 | | |

*$p < .05$, **$p < .01$

***Mental state understanding (TOM scale).***

Hierarchical regression analyses predicting mental state understanding as assessed by the TOM scale (Table S10) revealed that age and gender accounted for approximately 5.8% of its variability, $F(2, 33) = 1.02$, $p = .372$. Introducing levels of social anxiety – assessed through levels of social worry and fear of negative evaluation – accounted for only an additional 2.9% of the variation in children's mental state understanding (TOM scale), and this change in $R^2$ was not significant, $F(2, 31) = .497$, $p = .613$. Adding levels of social avoidance accounted for zero additional variation in children's mental state understanding (TOM scale), and this change in $R^2$ was not significant, $F(1, 30) = .013$, $p = .909$.

**Table S10**

*Summary of Hierarchical Regression Analysis for gender, age, and social anxiety measures predicting mental state understanding (TOM scale)*.

| Predictor Variables | | *B* | β | *t* | $R^2$ | $\Delta R^2$ |
|---|---|---|---|---|---|---|
| Model 1 | | | | | .06 | .058 |
| | Age in months | .004 | .06 | .33 | | |
| | Gender of child | -.39 | -.25 | -1.43 | | |
| Model 2 | | | | | .09 | .029 |
| | Age in months | .001 | .01 | .07 | | |
| | Gender of child | -.49 | -.31 | -1.65 | | |
| | Social worry (SWAIY-M) | -.14 | -.17 | -.66 | | |
| | Fear of negative evaluation (BFNE) | .19 | .26 | .99 | | |
| Model 3 | | | | | .09 | .00 |
| | Age in months | .001 | .02 | .09 | | |
| | Gender of child | -.49 | -.32 | -1.61 | | |
| | Social worry (SWAIY-M) | -.13 | -.17 | -.63 | | |
| | Fear of negative evaluation (BFNE) | .19 | .27 | .98 | | |
| | Social Avoidance (SWAIY-A) | -.02 | -.02 | -.12 | | |

$^*p < .05$, $^{**}p < .01$

***Mental state understanding (CSUS).***

Hierarchical regression analyses predicting mental state understanding as assessed by the CSUS parent-report (Table S11) revealed that age and gender accounted for approximately 9.9%

of its variability, $F(2, 33) = 1.82$, $p = .178$. Introducing levels of social anxiety – assessed through levels of social worry and fear of negative evaluation – accounted for an additional 2.2% of the variation in children's mental state understanding (CSUS), and this change in $R^2$ was not significant, $F(2, 31) = .384$, $p = .684$. Adding levels of social avoidance accounted for only an additional 1.8% of children's mental state understanding (CSUS), and this change in $R^2$ was not significant, $F(1, 30) = .637$, $p = .431$.

**Table S11**

*Summary of Hierarchical Regression Analysis for gender, age, and social anxiety measures predicting mental state understanding (CSUS).*

| Predictor Variables | | *B* | β | *t* | $R^2$ | $\Delta R^2$ |
|---|---|---|---|---|---|---|
| Model 1 | | | | | .01 | .099 |
| | Age in months | -.03 | -.33 | -1.90 | | |
| | Gender of child | .14 | .07 | .38 | | |
| Model 2 | | | | | .12 | .022 |
| | Age in months | -.03 | -.33 | -1.81 | | |
| | Gender of child | .12 | .06 | .31 | | |
| | Social worry (SWAIY-M) | -.16 | -.14 | -.57 | | |
| | Fear of negative evaluation (BFNE) | -.007 | -.007 | -.03 | | |
| Model 3 | | | | | .14 | .018 |
| | Age in months | -.03 | -.36 | -1.92 | | |
| | Gender of child | .18 | .09 | .45 | | |
| | Social worry (SWAIY-M) | -.19 | -.17 | -.66 | | |

| | | | | | | |
|---|---|---|---|---|---|---|
| | Fear of negative evaluation (BFNE) | -.05 | -.05 | -.19 | | |
| | Social Avoidance (SWAIY-A) | .15 | .15 | .798 | | |

*p < .05, **p < .01

***Social perspective taking (FIMI).***

Hierarchical regression analyses predicting child-reported social perspective taking (Table S12) revealed that age and gender accounted for 1.6% of its variability, $F(2, 33) = .269$, $p = .766$. Introducing levels of social anxiety – assessed through levels of social worry and fear of negative evaluation – accounted for an additional 5.4% of the variation in children's self-reported perspective taking (FIMI), and this change in $R^2$ was not significant, $F(2, 31) = .895$, $p = .419$. Adding levels of social avoidance accounted for an additional 8.5% of the variation in children's self-reported perspective taking (FIMI), and this change in $R^2$ approached significance, $F(1, 30) = 3.03$, $p = .092$.

**Table S12**

*Summary of Hierarchical Regression Analysis for gender, age, and social anxiety measures predicting a child's self-reported social perspective taking (FIMI).*

| Predictor Variables | | *B* | β | *t* | $R^2$ | $\Delta R^2$ |
|---|---|---|---|---|---|---|
| Model 1 | | | | | .02 | .016 |
| | Age in months | -.008 | -.10 | -.57 | | |
| | Gender of child | 194 | .11 | .59 | | |
| Model 2 | | | | | .07 | .054 |
| | Age in months | -.004 | -.04 | -.24 | | |

| | | | | | | |
|---|---|---|---|---|---|---|
| | Gender of child | .34 | .18 | .96 | | |
| | Social worry (SWAIY-M) | .16 | .16 | .64 | | |
| | Fear of negative evaluation (BFNE) | -.28 | -.34 | -1.26 | | |
| Model 3 | | | | | .16 | .085 |
| | Age in months | .002 | .03 | .14 | | |
| | Gender of child | .23 | .12 | .66 | | |
| | Social worry (SWAIY-M) | .21 | .22 | .87 | | |
| | Fear of negative evaluation (BFNE) | -.21 | -.25 | -.92 | | |
| | Social Avoidance (SWAIY-A) | -.28 | -.33 | -1.74 | | |

*$p < .05$, **$p < .01$

**Discussion**

In our exploratory analyses comparing the younger (6-8 years) and older (9-12 years) subgroups of our sample we found that the significant relationships between fear of negative evaluation and mental state understanding (i.e., positively related to the 2-item behavioural TOM task and negatively related to the parent-report CSUS), were limited to the younger half of the sample, with the relationship between fear of negative evaluation and the parent-report measure of mental state understanding significant after False Discovery Rate correction. This could suggest that children who possess an early sensitivity to criticism, may be less inclined to consider the minds of others in their daily lives for fear of being negatively evaluated, which

could negatively impact their future social interactions and relationships. However, as this pattern did not hold in the older half of our sample, it is possible that, through social experience and exposure, children develop the necessary social skills so that the fear of negative evaluation no longer affects their ability (or willingness) to reason about the minds of others. It may be important for clinicians working with young children to address misconceptions surrounding the judgements of others to support social cognitive development. For example, interventions aimed at reducing fears of negative evaluation may prove especially helpful. Future research using a longitudinal design could also examine whether early developing social anxiety predicts or interacts with later social cognitive processing and how those influence children's social skills and difficulties over time.

Conversely, the negative relationship between social avoidance and self-reported social perspective taking (FIMI) was only found in the older half of the sample. This finding raises the idea that social avoidance has a cumulative effect on certain components of social cognition (Hu, 2024), such that its influence is potentially stronger, or more likely to be observed, in older children. This aligns with prior work indicating social avoidance as a key predictor and maintenance of social anxiety (Ghandchi et al., 2023; McClure & Nowicki, 2001; Rodebaugh et al., 2025). However, these age-related findings warrant cautious interpretation, as splitting the original sample into two smaller groups limits the statistical power and reliability of these analyses. Future research would benefit from replications in targeted age samples and longitudinal designs.

**Supplementary Section S4:** ***Variance Inflation Factor values***

**Regression predicting prosodic emotion recognition**

| **Variable** | **Tolerance** | **VIF** |
|---|---|---|

| | | |
|---|---|---|
| Age (in months) | .881 | 1.135 |
| Gender | .986 | 1.014 |
| Social Worry | .610 | 1.639 |
| Social Anxiety | .598 | 1.672 |
| Social Avoidance | .772 | 1.295 |

**Regression predicting 2-item TOM scale**

| Variable | Tolerance | VIF |
|---|---|---|
| Age (in months) | .881 | 1.135 |
| Gender | .986 | 1.014 |
| Social Worry | .610 | 1.639 |
| Social Anxiety | .598 | 1.672 |
| Social Avoidance | .772 | 1.295 |

**Regression predicting parent-report CSUS (mental state understanding)**

| Variable | Tolerance | VIF |
|---|---|---|
| Age (in months) | .889 | 1.124 |
| Gender | .989 | 1.011 |
| Social Worry | .613 | 1.630 |
| Social Anxiety | .599 | 1.670 |
| Social Avoidance | .774 | 1.292 |

**Regression predicting child-report social perspective taking**

| Variable | Tolerance | VIF |
|---|---|---|
| Age (in months) | .881 | 1.135 |
| Gender | .986 | 1.014 |
| Social Worry | .610 | 1.639 |
| Social Anxiety | .598 | 1.672 |
| Social Avoidance | .772 | 1.295 |
